# Meta-analysis of epigenome-wide association studies of major depressive disorder

**DOI:** 10.1038/s41598-022-22744-6

**Published:** 2022-11-01

**Authors:** Qingqin S. Li, Randall L. Morrison, Gustavo Turecki, Wayne C. Drevets

**Affiliations:** 1 Neuroscience, Janssen Research and Development, LLC, Titusville, NJ USA; 2 JRD Data Science, Janssen Research and Development, LLC, Titusville, NJ USA; 3 Douglas Mental Health University Institute, McGill University, Montreal, QC Canada; 4 Neuroscience, Janssen Research and Development, LLC, La Jolla, CA USA; 5 Present Address: RLM Consulting LLC, 200 S Landmark Lane, Fort Washington, PA 19034 USA

**Keywords:** Epigenetics in the nervous system, Epigenomics

## Abstract

Epigenetic mechanisms have been hypothesized to play a role in the etiology of major depressive disorder (MDD). In this study, we performed a meta-analysis between two case–control MDD cohorts to identify differentially methylated positions (DMPs) and differentially methylated regions (DMRs) in MDD. Using samples from two Cohorts (a total of 298 MDD cases and 63 controls with repeated samples, on average ~ 1.8 samples/subject), we performed an EWAS meta-analysis. Multiple cytosine-phosphate-guanine sites annotated to *TNNT3* were associated with MDD reaching study-wide significance, including cg08337959 (*p* = 2.3 × 10^–11^). Among DMPs with association *p* values less than 0.0001, pathways from REACTOME such as Ras activation upon Ca^2+^ influx through the NMDA receptor (*p* = 0.0001, p-adjusted = 0.05) and long-term potentiation (*p* = 0.0002, p-adjusted = 0.05) were enriched in this study. A total of 127 DMRs with Sidak-corrected *p* value < 0.05 were identified from the meta-analysis, including DMRs annotated to *TNNT3* (chr11: 1948933 to 1949130 [6 probes], Sidak corrected *P* value = 4.32 × 10^–41^), *S100A13* (chr1: 153599479 to 153600972 [22 probes], Sidak corrected *P* value = 5.32 × 10^–18^), *NRXN1* (chr2: 50201413 to 50201505 [4 probes], Sidak corrected *P* value = 1.19 × 10^–11^), *IL17RA* (chr22: 17564750 to 17565149, Sidak corrected *P* value = 9.31 × 10^–8^), and *NPFFR2* (chr4: 72897565 to 72898212, Sidak corrected *P* value = 8.19 × 10^–7^). Using 2 Cohorts of depression case–control samples, we identified DMPs and DMRs associated with MDD. The molecular pathways implicated by these data include mechanisms involved in neuronal synaptic plasticity, calcium signaling, and inflammation, consistent with reports from previous genetic and protein biomarker studies indicating that these mechanisms are involved in the neurobiology of depression.

## Introduction

Previous genome-wide association studies and integrated genomic analysis have implicated the roles of synaptic structure especially excitatory synaptic pathways, neurotransmission, calcium signaling, and frontal brain region in depression^1–3^. In addition to genetic mechanisms, epigenetic mechanisms that alter chromatin structure and/or modulate gene expression patterns also play a role in the disease etiology^4^. Early life adversity is a major risk factor for major depressive disorder (MDD) and influences crosstalk among multiple mechanisms of genomic regulation, including histone marks, DNA methylation, and the transcriptome^5,6^. An increase in histone H3 acetylation and decrease in histone deacetylase 2 (HDAC2) in the nucleus accumbens, a limbic brain region implicated in reward processing, was reported in both preclinical mouse model of depression (chronic social defeat stress paradigm) and post-mortem brain of depressed patients^7^. Infusion of HDAC inhibitors into the nucleus accumbens increases histone acetylation and exerts antidepressant-like effects in the social defeat stress paradigm, which is accompanied by a reversal of gene expression pattern induced by chronic social defeat and mimicking the effect of antidepressant fluoxetine^7^. Another mechanism of epigenetic regulation is DNA methylation which also regulates gene expression changes. Genome-wide changes in the DNA methylation pattern reflect complex interactions between environment and genetics^8^. Epigenome-wide association study (EWAS), also known as Methylome-wide association study (MWAS), is a promising complement to genome-wide association study (GWAS) and chromatin remodeling by histone acetylation.

Aberg et al. conducted an EWAS study using methyl-CG binding domain sequencing (MBD-Seq) and MDD cases and controls from both blood (N = 1132) and postmortem brain tissues (N = 61 samples from the medial prefrontal cortical region of Brodmann Area 10 [BA10]), and showed significant overlap (*p* = 5.4 × 10^–3^) between the EWAS findings in blood and brain (i.e., BA10)^9^. Several EWAS studies of MDD have been conducted using blood samples. EWAS using blood samples comparing current vs. never MDD status was performed among World Trade Center responders (trauma-exposed)^10^. Clark et al. conducted an MWAS using MBD-Seq and 581 blood samples with current MDD at baseline and assess the profile with current MDD diagnosis status in year 6 and identified themes on cellular responses to stress and signaling mechanisms linked to immune cell migration and inflammation^11^. Postpartum depression (PPD) was also studied including both prepartum euthymic and prepartum depressed samples and a cross-species translational mouse model (17β-estradiol (E2)) which implicated hippocampal synaptic plasticity in PPD^12^. A small pilot study was also performed to study ECT response (n = 12)^13^. The largest EWAS study to date used self-reported antidepressant use as a surrogate for depression and used 6,428 samples from the Generation Scotland (GS) database and 2449 samples from the Netherlands Twin Registry and identified ten DMPs in the GS Cohort but only one of these DMPs was statistically confirmed in the meta-analysis between these two Cohorts^14^. In contrast, few EWAS studies have been conducted in brain tissue samples from depressed patients studied postmortem. One other EWAS study using brain samples for late-life depression status was conducted using brain samples from the ROSMAP Cohort^15^. A table summarizing the previously reported EWAS is provided in Supplementary Table 1.

Inspired by the brain-blood correlation, we set out to perform EWAS in two MDD Cohorts and performed a meta-analysis between these two Cohorts. Both DMPs and DMRs were identified, and the results were discussed in the context of enriched pathways.

## Results

### Differentially methylated positions in peripheral blood

**Figure 1:**
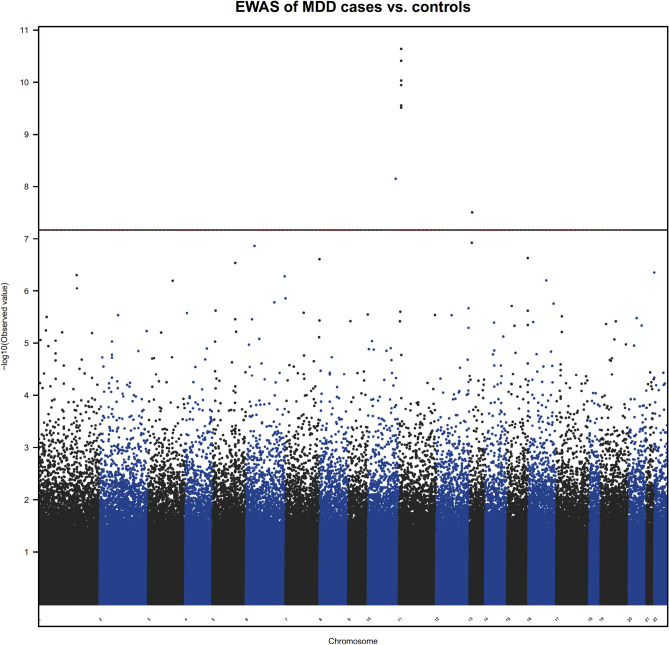
Manhattan plot of DMP analysis of MDD case–control EWAS meta-analysis.

**Figure 2:**
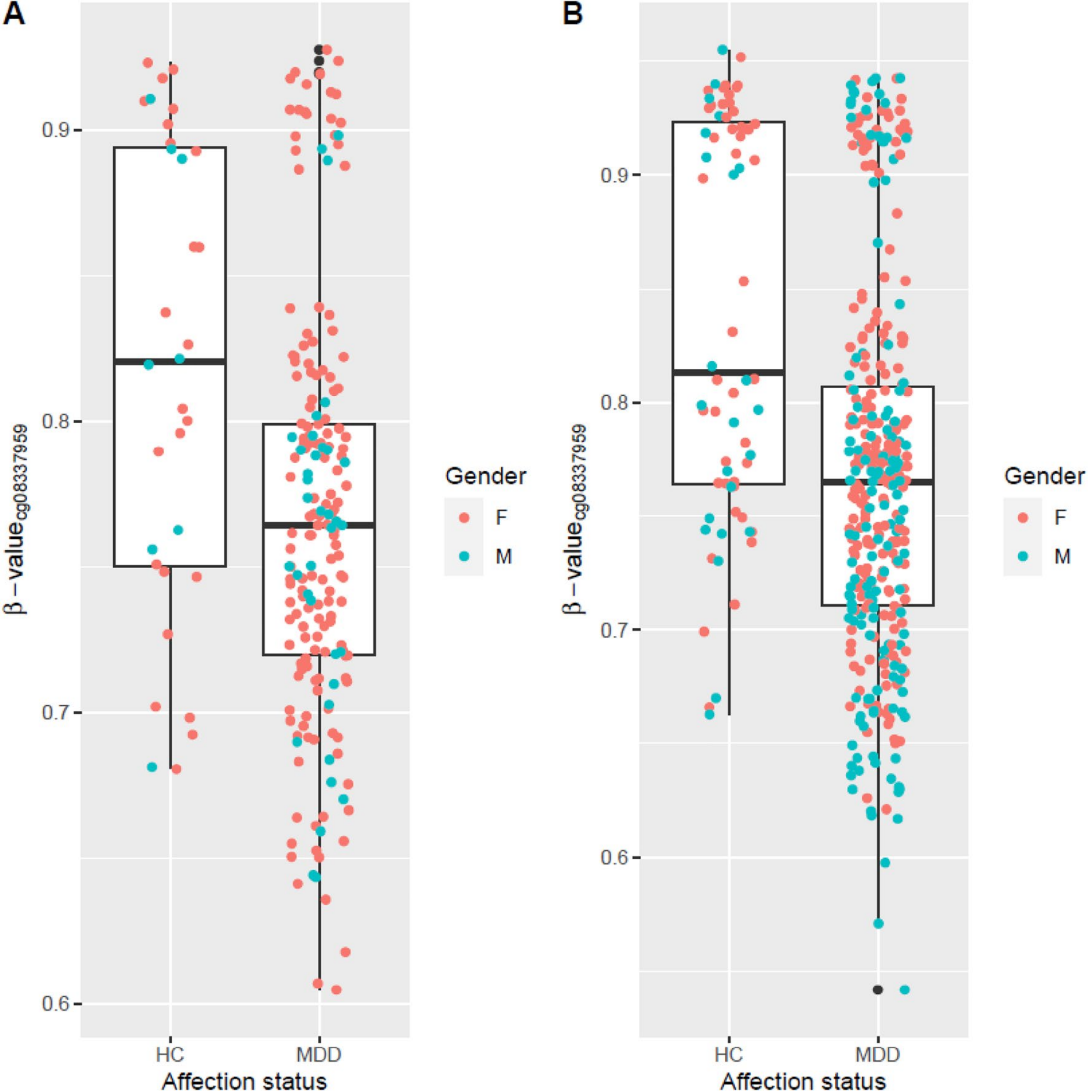
Association of cg08337959 annotated to *TNNT3* with MDD case–control status in (**A**) cohort 1, (**B**) cohort 2.

Samples used in the EWAS analyses were described in Supplementary Table 2. 78.5% of the MDD cases and 34.4% of the healthy controls from cohort 1 were of European ancestry. In cohort 2, 79.7% of the MDD cases and 79.4% of the healthy controls from cohort 1 were of European ancestry. In the EWAS meta-analysis between the two cohorts, eight CpG sites including six annotated to *TNNT3* (cg08337959 *p* = 2.29 × 10^–11^, cg01821149 *p* = 3.06 × 10^–10^), were associated with MDD case status passing Bonferroni correction threshold (*p* <  = 0.05/740, 121 ~ 6.76 × 10^–8^, Supplementary Fig. 1A [Cohort 1 Q-Q plot], 2A [Cohort 1 Manhattan plot], genomic inflation factor lambda (l_cohort1_) = 1.048; Supplementary Fig. 1B [Cohort 2 Q–Q plot], 2B [Cohort 2 Manhattan plot], genomic inflation factor lambda (l_cohort2_) = 1.115; Fig. 1 [Meta-analysis Manhattan plot], Table 1, Supplementary Fig. 1C [Meta-analysis Q–Q plot]). cg08337959 was associated with MDD in both cohort 1 (b = − 0.52, *p* = 2.37 × 10^–4^) and cohort 2 (b = − 0.71, *p* = 2.20 × 10^–8^). Hypomethylation of CpG sites annotated to *TNNT3* was associated with MDD case status in both cohorts (Fig. 2, cg08337959, b = − 0.52, *p* = 2.37 × 10^–4^ in cohort 1 and b = − 0.71, *p* = 6.46 × 10^–8^ in cohort 2; Supplementary Fig. 3, cg01821149 b = − 0.24, *p* = 7.15 × 10^–4^ in cohort 1 and b = − 0.27, *p* = 1.02 × 10^–7^ in cohort 2, Table 1) and these associations remained unchanged after correcting the CpG probe cg05575921 annotated to *AHRR*, which serves as a surrogate for smoking status. Corrections for genetic population substructure or medication status did not significantly change the significance. A full list of DMPs with *p* value less than study wide significance threshold in individual cohorts or *p* value < 1 × 10^–6^ in the meta-analysis are provided in Supplementary Tables 3 and 4, respectively.

**Table 1:** MDD EWAS meta-analysis genome wide significant CpG sites.

chr	pos	Name	GencodeCompV12_NAME	pval	beta	se	k	I^2^	tau2	Cohort 1					Cohort 2				
										*M-value*		*b-value*		M-value	*M-value*		*b-value*		M-value
										beta	AveMethylation	beta	AveMethylation	*P* value	beta	AveMethylation	beta	AveMethylation	*P* value
chr11	1948933	cg08337959	*TNNT3*	2.29E−11	− 0.74	0.11	2	0	0	− 0.52	1.86	− 0.05	0.77	2.37 E−04	− 0.71	1.98	− 0.06	0.78	2.21 E−08
chr11	1949130	cg15652404	*TNNT3*	3.87 E−11	− 0.73	0.11	2	0	0	− 0.43	1.14	− 0.05	0.68	2.73 E−04	− 0.47	1.14	− 0.07	0.68	3.27 E−08
chr11	1949113	cg18032502	*TNNT3*	9.24 E−11	− 0.72	0.11	2	0	0	− 0.49	1.83	− 0.05	0.77	5.21 E−04	− 0.67	1.98	− 0.06	0.78	4.11 E−08
chr11	1949039	cg06503573	*TNNT3*	1.13 E−10	− 0.71	0.11	2	0	0	− 0.49	1.28	− 0.06	0.70	8.53 E−05	− 0.48	1.27	− 0.06	0.70	2.89 E−07
chr11	1949032	cg06679296	*TNNT3*	2.79 E−10	− 0.70	0.11	2	0	0	− 0.51	2.35	− 0.04	0.82	1.00 E−03	− 0.70	2.51	− 0.05	0.83	6.46 E−08
chr11	1949090	cg01821149	*TNNT3*	3.06 E−10	− 0.70	0.11	2	0	0	− 0.24	0.75	− 0.03	0.63	7.15 E−04	− 0.27	0.63	− 0.04	0.61	1.02 E−07
chr10	129653554	cg17210803		7.08 E−09	− 0.64	0.11	2	0	0	− 0.08	0.19	− 0.01	0.53	4.29 E−03	− 0.11	0.21	− 0.02	0.54	3.92 E−07
chr13	30689528	cg11283819		3.11 E−08	0.61	0.11	2	0	0	0.14	− 1.69	0.02	0.24	4.99 E−04	0.14	− 1.79	0.02	0.23	1.49 E−05

beta, estimated coefficients of the model; k, number of studies included in the analysis; pval, corresponding *p* values; tau2, estimated amount of (residual) heterogeneity; I^2^ statistics, estimates (in percent) how much of the total variability in the observed effect sizes (which is composed of heterogeneity plus sampling variability) can be attributed to heterogeneity among the true effects

Among the ten CpG sites significantly associated with self-reported antidepressant use reported from the Generation Scotland cohort^14^, two of them (cg03864397 annotated to *CASP10* implicated in innate immune response, b = − 0.23, *p* = 0.03; cg26277237 annotated to *KANK1*, b = 0.24, *p* = 0.03) were nominally significant in this meta-analysis (*p* < 0.05) and with consistent direction in effect size (Supplementary Table 5). The correlation of effect size/beta coefficient between the overlapping CpG sites (70 for cohort 1 and 71 for cohort 2) used for methylation score calculation^14^ and the individual cohort was insignificant, but the directionality was consistent for cohort 2 (r = 0.15, *p* = 0.21, Supplementary Fig. 4). The methylation score calculated using the same overlapping CpG sites weighted by the effect size did not distinguish cases from controls in a linear mixed model (b = 0.013, *p* = 0.18) but the directionality was consistent in cohort 2. For cohort 1, the methylation score for MDD was lower than controls (b = − 0.018, *p* = 0.04), suggesting that the weights of the methylation score could benefit from an even bigger study or EWAS meta-analysis in the future.

### Pathway enrichment analysis

**Figure 3:**
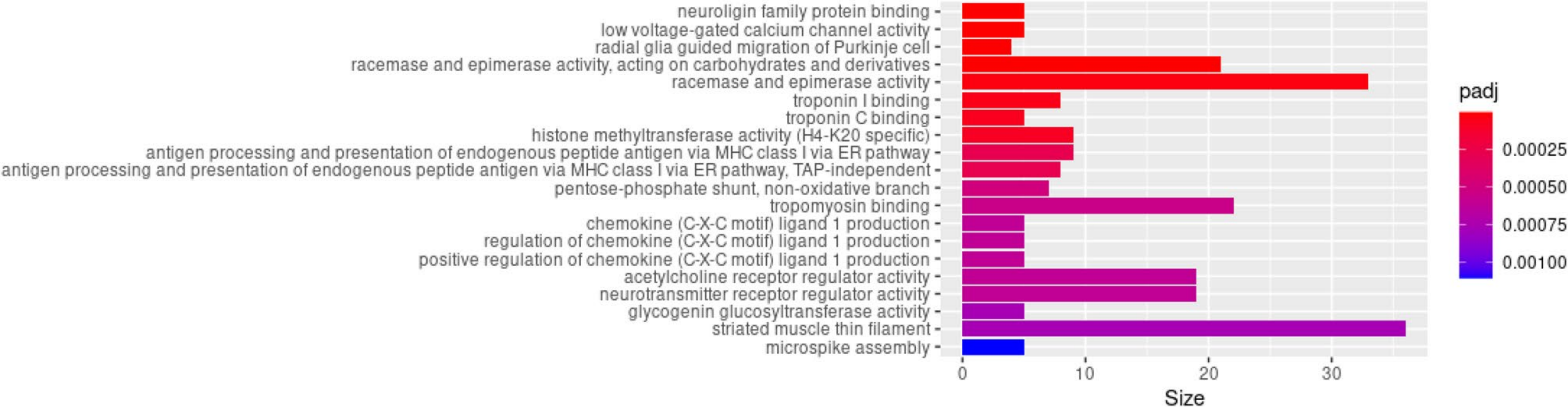
Enriched gene sets from the GO database (min gene set size = 4) among DMP associated with MDD case status with a *p* value less than 1 × 10^–4^ in the EWAS meta-analysis.

Pathway enrichment analysis using logistic regression adjusting for the number of CpG sites per gene on the EPIC arrays using methylglm from methylglm and DMP with association *p* value less than 1 × 10^–4^ revealed enrichment of neuroligin family protein binding (*p* = 1.30 × 10^–36^, adjusted *p* value = 1.78 × 10^–32^), low voltage-gated calcium channel activity (*p* = 1.99 × 10^–16^, adjusted *p* value = 1.37 × 10^–12^), chemokine (C-X-C motif) ligand 1 production (*p* = 7.53 × 10^–7^, adjusted *p* value = 6.14 × 10^–4^) (Fig. 3 and Supplementary Table 6).

### Differentially methylated region (DMR) analysis

**Figure 4:**
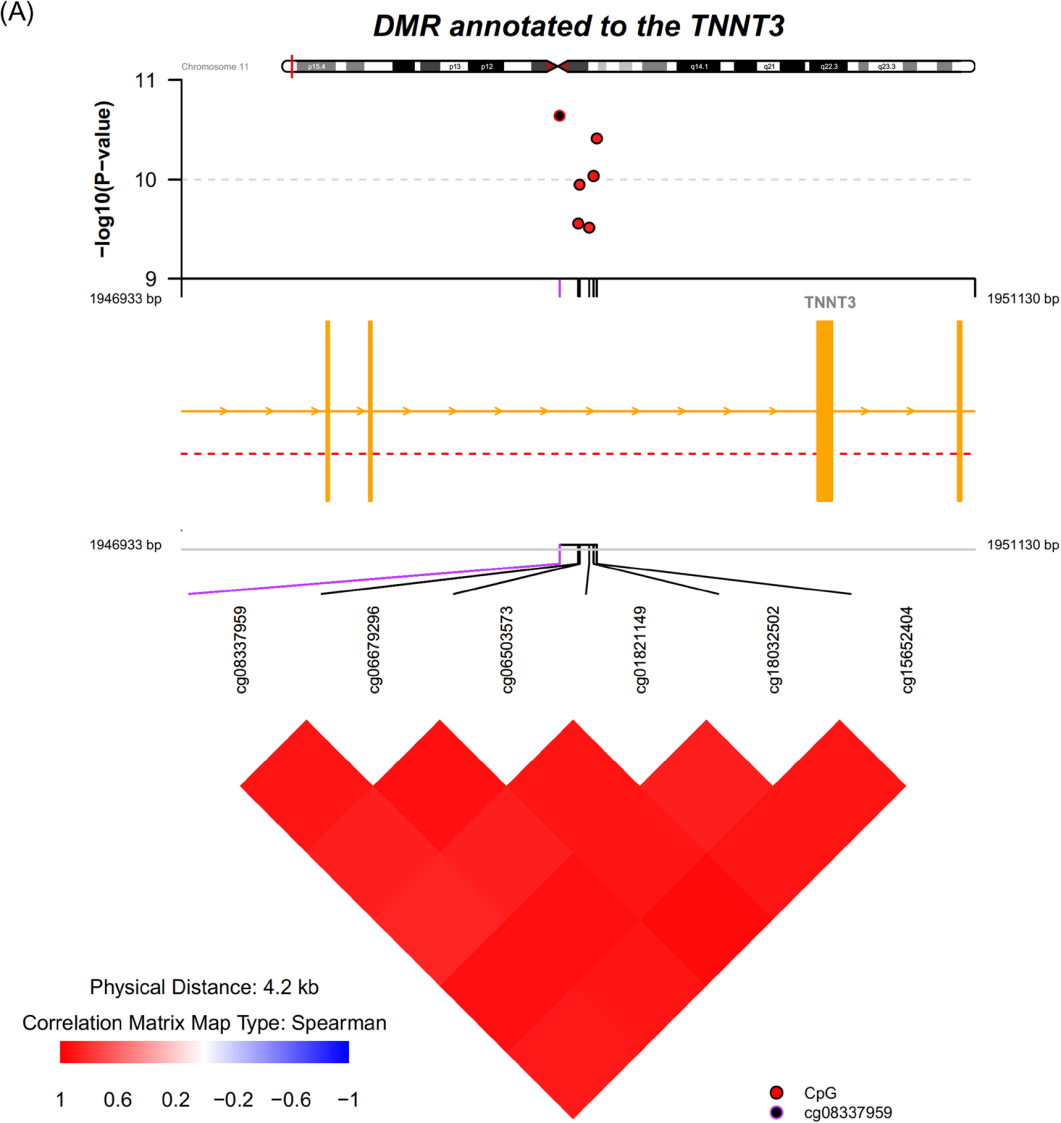

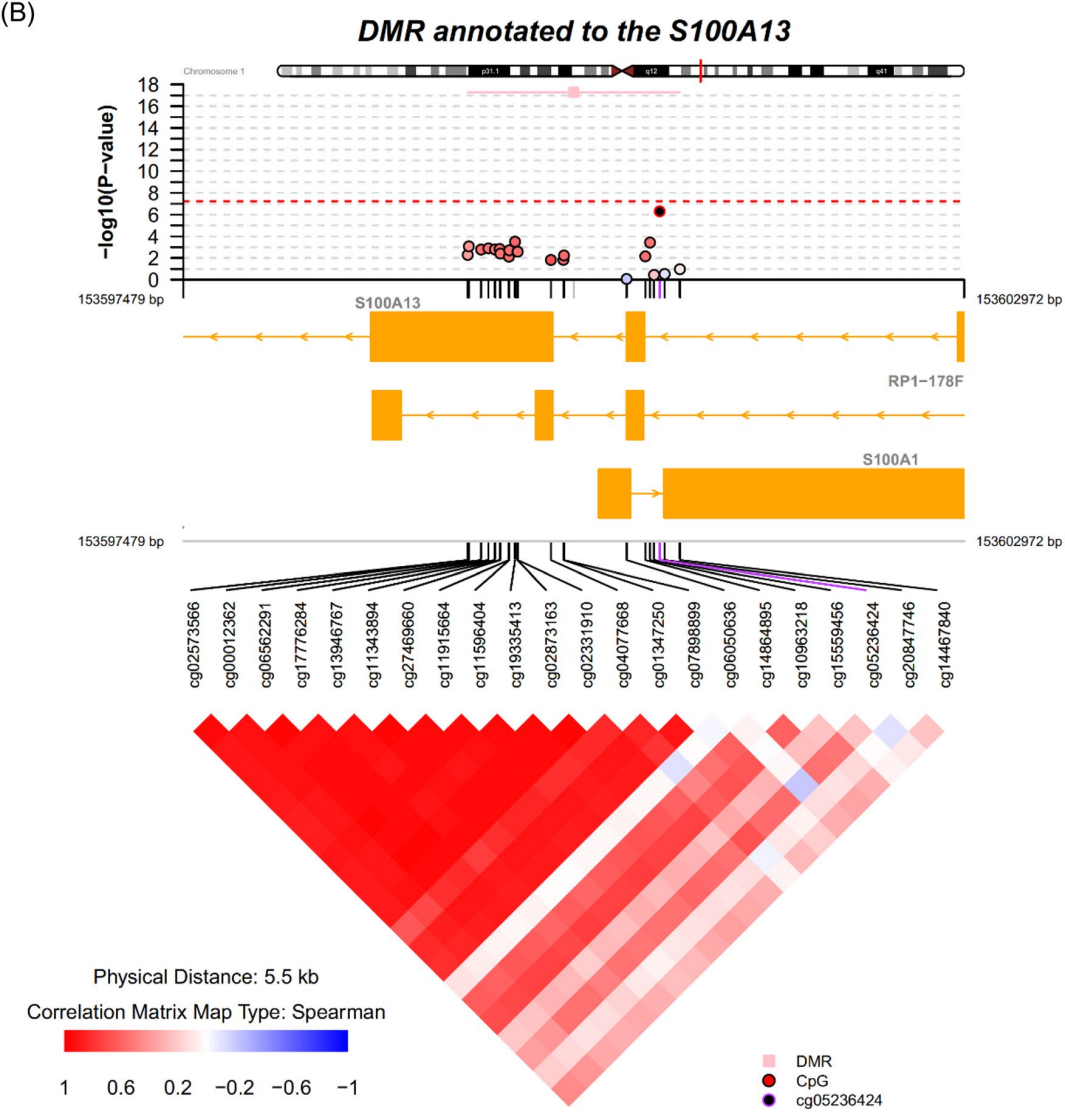

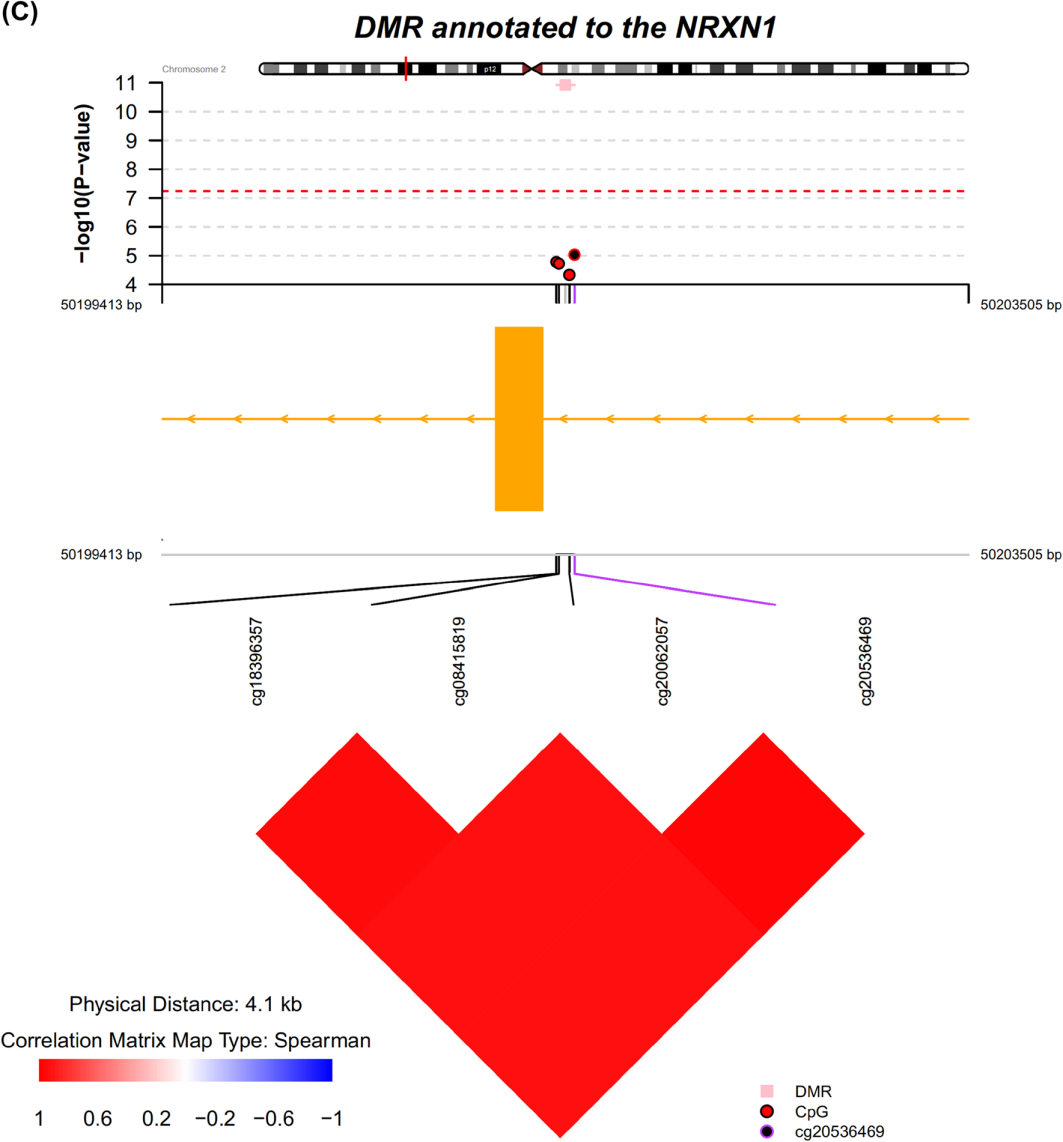
DMR annotated to *TNNT3* (**A**), *S100A13* (**B**)*,* and *NRXN1* (**C**) association with the MDD case–control status. Top panel: individual CpG association *P* values; middle panel: gene structure; bottom panel: pairwise correlation between CpG sites in this DMR. CpG, cytosine-phosphate-guanine; DMR, differentially methylated region.

DMR analysis enabled the identification of regions in the genome consisting of ≥ 3 probes with consistent signals associated with MDD. Overall, the analyses performed using the comb-p algorithm identified 127 DMRs as being significantly associated with MDD at the Sidak-corrected *p* value < 0.05 from the meta-analysis. These results included DMRs annotated to *TNNT3* (chr11: 1,948,933 to 1,949,130 [6 probes], Sidak corrected *P* value = 4.32 × 10^–41^, Fig. 4A), S100 calcium-binding protein A13 (*S100A13*, chr1: 153599479 to 153600972 [22 probes], Sidak corrected *P* value = 5.32 × 10^–18^, Fig. 4B), neurexin 1 (*NRXN1*) (chr2: 50201413 to 50201505 [4 probes], Sidak corrected *P* value = 1.19 × 10^–11^, Fig. 4C), interleukin 17 receptor A (*IL17RA*) (chr22: 17564750 to 17565149, Sidak corrected *P* value = 9.31 × 10^–8^, Supplementary Fig. 5), and neuropeptide FF receptor 2 (*NPFFR2*) (chr4: 72897565 to 72898212, Sidak corrected *P* value = 8.19 × 10^–7^). For *IL17RA*, one of the CpG sites cg07191900 giving rise to the DMR was hypermethylated in MDD (b = 0.55, *p* = 4.41 × 10^–7^), while two other CpG sites were likewise hypermethylated (cg20758542 b = 0.45, *p* = 4.62 × 10^–5^; cg13595439 b = 0.21, *p* = 0.06). A full list of DMRs with Sidak-corrected *p* value < 0.05 is available in Supplementary Table 7. The probes underlying each DMR are also provided in Supplementary Table 8.

## Discussion

In the meta-analysis across two cohorts, CpG sites annotated to *TNNT3* passed the genome-wide significance threshold. There was no prior report linking *TNNT3* to psychiatry conditions. We additionally identified 127 DMRs associated with MDD, among which several of the implicated genes warrant additional discussion.

Among the DMPs associated with MDD in the meta-analysis, neuroligin family protein binding was among the top pathways enriched in this study. These proteins of the neuroligin family are neuronal cell surface proteins. They act as splice site-specific ligands for b-neurexins and may be involved in synaptogenesis. Neurexin 1 variants were previously implicated as risk factors for suicide death based on shared chromosomal segment analysis^16^. A functional genomic experiment showed that two Neurexin variants increased binding to the postsynaptic binding partner *LRRTM2 in vitro*^17^. Other variants (SNVs and CNVs) in *NLGN1* and/or other family members *NLGN3* and *NLGN4* were previously associated with suicide, PTSD, autism, obsessive–compulsive disorder (OCD), and depression^18–25^. The variant rs6779753 in *NLGN1* underlying the gene-based PTSD association was also associated with the intermediate phenotypes of higher startle response and greater functional magnetic resonance imaging (fMRI) activation of various brain regions including the amygdala and orbitofrontal cortex in response to fearful face. A rare variant in *NLGN1* was also implicated in autism^26^*.* Presynaptic *NRXN1*^22,27,28^, *NRXN2*, *NRXN3*, and cytoplasm partners *SHANK1*^29^, *SHANK2*^30^, *SHANK3*^31,32^, *EPAC*^33^, *MDGA*^34^, *DLG4*, and *DLGAP2* were also implicated in autism^35^, mental retardation^30^, and/or schizophrenia^28^. Overall, there is substantial genetic evidence implicating the NRXN-NLGN pathway in suicide and other psychiatric conditions. In addition, transcriptional activity in neurexin and neuroligin genes is regulated by methylation^36^. Sleep deprivation has caused a shift in methylation patterns in both neurexin and neuroligin in animals^37^. *NLGN1* was also implicated in the animal model of depression^38^. Herein we additionally provide epigenetic evidence in the involvement of this pathway by demonstrating differentially methylated region in *NRXN1* in peripheral blood and the pathway enrichment of neuroligin family protein binding.

Among the other DMRs associated with MDD, *S100A13* plays a role in the central nervous system (CNS) development and it is especially expressed in the developing human hippocampus and temporal cortex^39^ and is differentially expressed in the orbitofrontal cortex of suicide victims^40^. Neuroinflammation and T-helper 17 (Th17) cells and IL17-A have been implicated in depression^41^. Th17 cells increased in preclinical depression animal models (learned helplessness and chronic restraint stress paradigms) and blockage of Th17 cell differentiation by a deficiency in retinoic acid receptor-related orphan receptor (ROR)γT transcription factor and inhibition of RORγT transcription factor pharmacologically or using IL17-A antibody rendered the animal resistant to learned helplessness^42^. IL1b and TGFb are required for Th17 cell differentiation and Th17 cells produce IL17, IL21, and IL22. Both TGFb and IL17 levels were reported to be elevated in depressed patients in a small study (41 MDD patients vs. 40 healthy controls)^43^. Another small study (40 MDD patients and 30 healthy controls) also showed increased peripheral Th17 cell count and reduced T-reg cell count (hence imbalance of Th17/Treg ratio), higher mRNA level of RORγT transcription factor, and increased serum IL17 in MDD patients compared to healthy controls^44^. *IL17RA* encodes a low-affinity receptor for IL17A. IL17A and its receptor could play a pathogenic role in many inflammatory and autoimmune diseases including multiple sclerosis, autism spectrum disorders, epilepsy, Alzheimer’s disease, and rheumatoid arthritis^41^. We herein provide epigenetic evidence for a DMR annotated to *IL17RA* although the underlying probes were hypermethylated in MDD, which could result in down-regulation of *IL17RA*. This could be a reflection of a compensatory mechanism to combat inflammation in MDD. Other inflammatory pathways such as chemokine (C-X-C motif) ligand 1 production were also enriched in this study.

There are several limitations of this study that merit comment. First, the healthy control sample size is relatively small despite the sample size for MDD cohorts being moderate. Secondly, the MDD cases were pooled together for meta-analysis to enhance the power, but there was heterogeneity between cohort 1 and cohort 2. Cohort 1 came from a naturalistic longitudinal follow-up study where samples at baseline were recently responding to antidepressant treatment (within three months), but approximately one-quarter of the samples relapsed during the follow-up period. Cohort 2 came from an antidepressant treatment study, and therefore samples at baseline were acutely ill, and samples at week 8 may or may not be responding to the treatment. In addition, although we attempted to control for medication status as a sensitivity analysis and the kind of medication exposed in cohort 2, we cannot rule out there is an influence of methylation status from prior medication exposure despite there was a wash-out period prior to subsequent treatment exposure. Treatment naïve samples would be in a better position to address this caveat. Lastly, early life adversity is known to influence DNA methylation. It is possible that the surrogate variables included in the statistical model capture some aspects of the systematic changes induced by early-life adversity. Systematically collecting this environmental factor for direct modeling of early life adversity in the epigenetic analysis will enable interaction analysis to study the impact of disease pathology and environmental factor simultaneously. Future epigenetic studies and meta-analyses with other MDD cohorts in the scientific community will further elucidate the epigenetic mechanisms associated with depression.

## Methods

### Study cohorts

#### Cohort 1

A total of 191 blood samples from 112 patients with MDD were collected up till the interim analysis from an observational clinical study OBSERVEMDD0001 (ClinicalTrials.gov Identifier: NCT02489305), where a patient must have met DSM-V criteria for nonpsychotic, recurrent MDD within the past 24 months (ie, the start of the most recent major depressive episode (MDE) must be ≤ 24 months before screening); have a Montgomery Asberg Depression Rating Scale (MADRS) total score ≤ 14 at screening and baseline visits; have evidence of recent response (within the past 3 months) to an oral antidepressant treatment regimen (taken at an optimal dosage and for an adequate duration, and be currently taking and responding to an oral antidepressant treatment regimen. The samples from the participants with MDD could have been obtained from either a baseline visit or a follow-up visit. 32 samples from 32 healthy controls self-reported to be free of MDD were collected by BioIVT and used as control samples for this Cohort. The institutional review boards of all participating clinical trial sites reviewed and approved the study and patients provided informed consent for DNA sample collection.

#### Cohort 2

A total of 359 MDD samples from 186 patients were drawn from the Molecular Biomarkers of Antidepressant Response study^45,46^, where a patient must have had a diagnosis of a current major depressive episode (MDE) as per the SCID-I and Hamilton Rating Scale for Depression (HAMD-21) ≥ 20, while 68 control samples from 31 patients were recruited through advertisement. Two or more samples from the same patient could have been collected (Supplementary Table 2).

The OBSERVEMDD0001 study was approved by the respective local or central Institutional Review Boards (IRBs) overseeing the clinical sites participating in the study, these included the University of Pennsylvania Office of Regulatory Affairs IRB, University of Iowa IRB, Baylor College Of Medicine IRB, University of Michigan IRB, University of Cincinnati IRB, Sharp HealthCare IRB, Springfield Committee for Research Involving Human Subjects (SCRIHS), Western IRB, University of Kansas School of Medicine—Wichita Human Subjects Committee, Rush University Medical Center IRB, Hartford Hospital IRB, University of Massachusetts Medical School IRB, and Sterling IRB. In addition, the BioIVT samples were collected with IRB approval from Schulman IRB. Lastly, the Molecular Biomarkers of Antidepressant Response study was approved by Douglas Hospital Research Ethics Board. All clinical studies and sample collections were carried out following the ethical principles outlined in the Declaration of Helsinki and are consistent with Good Clinical Practices and applicable regulatory requirements. All patients provided written informed consent before entry into the study.

### Genotyping of samples

All samples from both cohorts were genotyped in a single batch using PsychArray (Illuminia, Inc., San Diego, CA). Standard QC was applied to remove samples with call rate less than 95%, variants with call rate less than 95%, minor allele frequency less than 1%, and variants deviating from Hardy–Weinberg equilibrium. Variants were thinned using PLINK v1.9^47,48^ using parameters “-indep-pairwise 1500 150 0.2” and variants in long-range linkage disequilibrium (LD) regions in chromosomes 6, 8, 5, and 11 reported previously were removed.^49^ The remaining variants were used to derive population substructure using eigenstrat v6.1.4^50,51^ using default parameters except adding the options of “nsnpldregress: 3 and maxdistldregress: 1” without outlier removal to preserve as many samples as possible since genetic ancestry does not seem to influence epigenetic profile significantly. The first two principal components were included as additional covariates in a sensitivity analysis described later.

### DNA methylation profiling

Whole blood samples were collected, and DNA was extracted for methylation profiling. DNA methylation was measured using Infinium® MethylationEPIC BeadChip (Illumina, Inc., San Diego, CA, USA) at 850 000 CpG sites throughout the genome. The assay for each cohort (both cases and controls) was performed in one batch. Genomic DNA samples were bisulfite-converted using the EZ-DNA Methylation Kits (Zymo Research, Irvine, CA, USA) and subsequently analyzed using the Illumina Infinium® HD methylation protocol on the HiScan™ system (Illumina, Inc, San Diego, CA, USA).

### Data pre-processing

Epigenetic data was analyzed separately for each Cohort. Quality control of the EPIC array data was performed using R package ChAMP^52^. Probes with detection *p* value ≥ 0.01 in one or more samples (n_Cohort1_ = 14,421 and n_Cohort2_ = 22,386, respectively), or with bead count less than 3 in at least 5% of samples (n_Cohort1_ = 8999 and n_Cohort2_ = 2104), non-CG probes (n_Cohort1_ = 2625 and n_Cohort2_ = 2586), probes with known SNP sites or with cross-reactivity^53^ (n_Cohort1_ = 93,722 and n_Cohort2_ = 93,024), probes align to multiple locations on the genome^54^ (n_Cohort1_ = 15 and n_Cohort2_ = 11), as well as probes located on the sex chromosomes (n_Cohort1_ = 16,532 and n_Cohort2_ = 16,186) were filtered out.

The methylation levels were normalized using the Dasen method in the R package wateRmelon^55^. The blood cell composition was estimated using the estimateCellCounts function in minfi^56^ which used a reference blood dataset of fluorescence-activated cell sorting (FACS) sorted CD8T, CD4T, NK, B cell, monocytes, granulocytes, and eosinophils^57^. Surrogate variables are covariates inferred from high-dimensional data that are used in subsequent analyses to adjust for unknown and/or unmodeled sources of noise^58,59^. We used R package sva (v3.38)^60,61^ to estimate surrogate variables for unknown sources of variation to remove artifacts in the epigenetic profile experiments. Removing batch effects using surrogate variables before downstream differential analysis has been shown to improve reproducibility^62^. One sample from cohort 2 with discrepant gender between case report form (CRF) and what was inferred based on epigenetic data was excluded from downstream analysis.

### Identification of DMPs

*M-value*, which provides higher detection rates and true positive rates for both highly methylated and unmethylated CpG sites and is considered statistically more valid than *beta-value*^63^, was used to identify DMPs using the R package limma^64^. However, the model fit using *beta-value* was also fit to report the effect size in *beta-value* only to ease biological interpretation*.* The primary analysis used the statistical model, adjusting for age, sex, cell composition, and surrogate variables (5 for cohort 1 and 10 for cohort 2) aiming to capture systematic technical variations was used to generate the contrast between MDD cases and healthy controls. Alternative statistical models additionally correcting for (1) smoking status using *AHRR* probe cg05575921^65^, (2) population substructure as represented by the first two principal components of the corresponding genetic data, (3) medication status (for the second cohort only) as an additional covariate was implemented as well as a sensitivity analysis. In all scenarios, sample relatedness was corrected by using the duplicateCorrelation function in limma. This was followed by a meta-analysis between the 2 Cohorts using the R package metafor^66^. DMPs with association *p* values less than the Bonferroni correction threshold (i.e. 0.05/number of CpG sites passing QC included in the analysis) were considered study-wide significant. The discovered DMPs were assessed for consistency in three ways: (1) the top DMPs discovered in a recent largest MWAS study^14^ were used to look for replication evidence from this study; (2) the effect sizes from this study were compared with the reported penalized regression coefficient^14^ from the full sample; (3) methylation score based on the same penalized regression coefficient^14^ was calculated and contrast between MDD cases and controls was assessed via a linear mixed model using R package lme4.

### Identification of DMRs

DMRs in the genome consisting of ≥ 3 probes were identified using comb-p^67^ with a distance of 500 bp and a seeded *p* value of 1.0 × 10^–4^. The DMRs with Sidak corrected *p* less than 0.05 were considered significant and reported.

### Gene set enrichment analysis

Gene set enrichment analysis was performed using methylglm function within R package methylGSA that accounts for length bias correction using logistic regression^68^ and DMPs with association *p* value less than 0.0001.Gene ontology databases used included KEGG database^69^ and c2.cp (a superset of BIOCARTA, KEGG, and REACTOME^70^ and a few other data sources) subsets of Molecular signatures database (MSigDB, v7.0)^71^.

## Supplementary Information

Supplementary Information 1.

Supplementary Information 2.

## Data Availability

Data used in the preparation of this article could be obtained from NCBI GEO under accession number GSE198904.

## References

[1] Howard DM (2019). Genome-wide meta-analysis of depression identifies 102 independent variants and highlights the importance of the prefrontal brain regions. Nat. Neurosci..

[2] Howard DM (2018). Genome-wide association study of depression phenotypes in UK Biobank identifies variants in excitatory synaptic pathways. Nat. Commun..

[3] Wingo TS (2021). Brain proteome-wide association study implicates novel proteins in depression pathogenesis. Nat. Neurosci..

[4] Fass DM, Schroeder FA, Perlis RH, Haggarty SJ (2014). Epigenetic mechanisms in mood disorders: Targeting neuroplasticity. Neuroscience.

[5] Lutz PE (2017). Association of a history of child abuse with impaired myelination in the anterior cingulate cortex: Convergent epigenetic, transcriptional, and morphological evidence. Am. J. Psychiatry.

[6] Lutz PE (2021). Non-CG methylation and multiple histone profiles associate child abuse with immune and small GTPase dysregulation. Nat. Commun..

[7] Covington HE (2009). Antidepressant actions of histone deacetylase inhibitors. J. Neurosci..

[8] Hoffmann A, Sportelli V, Ziller M, Spengler D (2017). Epigenomics of major depressive disorders and schizophrenia: Early life decides. Int. J. Mol. Sci..

[9] Aberg KA (2020). Methylome-wide association findings for major depressive disorder overlap in blood and brain and replicate in independent brain samples. Mol. Psychiatry.

[10] Kuan PF (2017). An epigenome-wide DNA methylation study of PTSD and depression in World Trade Center responders. Transl. Psychiatry.

[11] Clark SL (2020). A methylation study of long-term depression risk. Mol. Psychiatry.

[12] Guintivano J, Arad M, Gould TD, Payne JL, Kaminsky ZA (2014). Antenatal prediction of postpartum depression with blood DNA methylation biomarkers. Mol. Psychiatry.

[13] Moschny N (2020). Novel candidate genes for ECT response prediction-a pilot study analyzing the DNA methylome of depressed patients receiving electroconvulsive therapy. Clin. Epigenet..

[14] Barbu MC (2021). Methylome-wide association study of antidepressant use in Generation Scotland and the Netherlands Twin Register implicates the innate immune system. Mol. Psychiatry.

[15] Huls A (2020). Association between DNA methylation levels in brain tissue and late-life depression in community-based participants. Transl. Psychiatry.

[16] Coon H (2018). Genome-wide significant regions in 43 Utah high-risk families implicate multiple genes involved in risk for completed suicide. Mol. Psychiatry.

[17] William N (2021). Neurexin 1 variants as risk factors for suicide death. Mol. Psychiatry.

[18] Kilaru V (2016). Genome-wide gene-based analysis suggests an association between Neuroligin 1 (NLGN1) and post-traumatic stress disorder. Transl. Psychiatry.

[19] Jamain S (2003). Mutations of the X-linked genes encoding neuroligins NLGN3 and NLGN4 are associated with autism. Nat. Genet..

[20] Laumonnier F (2004). X-linked mental retardation and autism are associated with a mutation in the NLGN4 gene, a member of the neuroligin family. Am. J. Hum. Genet..

[21] Yan J (2005). Analysis of the neuroligin 3 and 4 genes in autism and other neuropsychiatric patients. Mol. Psychiatry.

[22] Glessner JT (2009). Autism genome-wide copy number variation reveals ubiquitin and neuronal genes. Nature.

[23] Lewis CM (2010). Genome-wide association study of major recurrent depression in the UK population. Am. J. Psychiatry.

[24] Gazzellone MJ (2016). Uncovering obsessive-compulsive disorder risk genes in a pediatric cohort by high-resolution analysis of copy number variation. J. Neurodev. Disord..

[25] Li Q (2021). 89. Genome wide meta-analysis of suicide behaviors. Eur. Neuropsychopharmacol..

[26] Nakanishi M (2017). Functional significance of rare neuroligin 1 variants found in autism. PLoS Genet..

[27] Bena F (2013). Molecular and clinical characterization of 25 individuals with exonic deletions of NRXN1 and comprehensive review of the literature. Am. J. Med. Genet. B Neuropsychiatr. Genet..

[28] Coelewij L, Curtis D (2018). Mini-review: Update on the genetics of schizophrenia. Ann. Hum. Genet..

[29] Sato D (2012). SHANK1 Deletions in males with autism spectrum disorder. Am. J. Hum. Genet..

[30] Berkel S (2010). Mutations in the SHANK2 synaptic scaffolding gene in autism spectrum disorder and mental retardation. Nat. Genet..

[31] Leblond CS (2014). Meta-analysis of SHANK mutations in autism spectrum disorders: A gradient of severity in cognitive impairments. PLoS Genet..

[32] Durand CM (2007). Mutations in the gene encoding the synaptic scaffolding protein SHANK3 are associated with autism spectrum disorders. Nat. Genet..

[33] Bacchelli E (2003). Screening of nine candidate genes for autism on chromosome 2q reveals rare nonsynonymous variants in the cAMP-GEFII gene. Mol. Psychiatry.

[34] Bucan M (2009). Genome-wide analyses of exonic copy number variants in a family-based study point to novel autism susceptibility genes. PLoS Genet..

[35] Peca J, Feng G (2012). Cellular and synaptic network defects in autism. Curr. Opin. Neurobiol..

[36] Runkel F, Rohlmann A, Reissner C, Brand SM, Missler M (2013). Promoter-like sequences regulating transcriptional activity in neurexin and neuroligin genes. J. Neurochem..

[37] Massart R (2014). The genome-wide landscape of DNA methylation and hydroxymethylation in response to sleep deprivation impacts on synaptic plasticity genes. Transl. Psychiatry.

[38] Feng P, Akladious AA, Hu Y (2016). Hippocampal and motor fronto-cortical neuroligin1 is increased in an animal model of depression. Psychiatry Res..

[39] Chan WY, Xia CL, Dong DC, Heizmann CW, Yew DT (2003). Differential expression of S100 proteins in the developing human hippocampus and temporal cortex. Microsc. Res. Technol..

[40] Thalmeier A (2008). Gene expression profiling of post-mortem orbitofrontal cortex in violent suicide victims. Int. J. Neuropsychopharmacol..

[41] Beurel E, Lowell JA (2018). Th17 cells in depression. Brain Behav. Immunol..

[42] Beurel E, Harrington LE, Jope RS (2013). Inflammatory T helper 17 cells promote depression-like behavior in mice. Biol. Psychiatry.

[43] Davami MH (2016). Elevated IL-17 and TGF-beta serum levels: A positive correlation between T-helper 17 cell-related pro-inflammatory responses with major depressive disorder. Basic Clin. Neurosci..

[44] Chen Y (2011). Emerging tendency towards autoimmune process in major depressive patients: A novel insight from Th17 cells. Psychiatry Res..

[45] Sun Y, Drevets W, Turecki G, Li QS (2020). The relationship between plasma serotonin and kynurenine pathway metabolite levels and the treatment response to escitalopram and desvenlafaxine. Brain Behav. Immun..

[46] Ju C (2019). Integrated genome-wide methylation and expression analyses reveal functional predictors of response to antidepressants. Transl. Psychiatry.

[47] Chang CC (2015). Second-generation PLINK: Rising to the challenge of larger and richer datasets. Gigascience.

[48] Purcell S (2007). PLINK: A tool set for whole-genome association and population-based linkage analyses. Am. J. Hum. Genet..

[49] Fellay J (2007). A whole-genome association study of major determinants for host control of HIV-1. Science.

[50] Patterson N, Price AL, Reich D (2006). Population structure and eigenanalysis. PLoS Genet..

[51] Price AL (2006). Principal components analysis corrects for stratification in genome-wide association studies. Nat. Genet..

[52] Morris TJ (2014). ChAMP: 450k chip analysis methylation pipeline. Bioinformatics.

[53] Zhou W, Laird PW, Shen H (2017). Comprehensive characterization, annotation and innovative use of infinium DNA methylation BeadChip probes. Nucleic Acids Res..

[54] Nordlund J (2013). Genome-wide signatures of differential DNA methylation in pediatric acute lymphoblastic leukemia. Genome Biol..

[55] Pidsley R (2013). A data-driven approach to preprocessing Illumina 450K methylation array data. BMC Genom..

[56] Aryee MJ (2014). Minfi: A flexible and comprehensive Bioconductor package for the analysis of Infinium DNA methylation microarrays. Bioinformatics.

[57] Guintivano J, Aryee MJ, Kaminsky ZA (2013). A cell epigenotype specific model for the correction of brain cellular heterogeneity bias and its application to age, brain region and major depression. Epigenetics.

[58] Leek JT, Storey JD (2007). Capturing heterogeneity in gene expression studies by surrogate variable analysis. PLoS Genet..

[59] Leek JT, Storey JD (2008). A general framework for multiple testing dependence. Proc. Natl. Acad. Sci. U.S.A..

[60] Leek JT, Johnson WE, Parker HS, Jaffe AE, Storey JD (2012). The sva package for removing batch effects and other unwanted variation in high-throughput experiments. Bioinformatics.

[61] Leek, J. T. *et al.**sva: Surrogate Variable Analysis* (R package version 3.30.1, 2019).

[62] Leek JT (2010). Tackling the widespread and critical impact of batch effects in high-throughput data. Nat. Rev. Genet..

[63] Du P (2010). Comparison of beta-value and M-value methods for quantifying methylation levels by microarray analysis. BMC Bioinform..

[64] Ritchie ME (2015). limma powers differential expression analyses for RNA-sequencing and microarray studies. Nucleic Acids Res..

[65] Tantoh DM (2020). AHRR cg05575921 methylation in relation to smoking and PM2.5 exposure among Taiwanese men and women. Clin. Epigenet..

[66] Viechtbauer W (2010). Conducting meta-analyses in R with the metafor package. J. Stat. Softw..

[67] Pedersen BS, Schwartz DA, Yang IV, Kechris KJ (2012). Comb-p: Software for combining, analyzing, grouping and correcting spatially correlated P-values. Bioinformatics.

[68] Mi G, Di Y, Emerson S, Cumbie JS, Chang JH (2012). Length bias correction in gene ontology enrichment analysis using logistic regression. PLoS ONE.

[69] Kanehisa M, Furumichi M, Tanabe M, Sato Y, Morishima K (2017). KEGG: New perspectives on genomes, pathways, diseases and drugs. Nucleic Acids Res..

[70] Fabregat A (2018). The Reactome Pathway Knowledgebase. Nucleic Acids Res..

[71] Liberzon A (2011). Molecular signatures database (MSigDB) 3.0. Bioinformatics.

