# Supplementary material for: Meta-analysis of epigenome-wide association studies of major depressive disorder
Source: Sci Rep. 2022 Nov 1;12:18361. doi: 10.1038/s41598-022-22744-6 (PMC9626569; doi:10.1038/s41598-022-22744-6)
Supplement: Supplementary file 2 — Supplementary Information 2. [file 41598_2022_22744_MOESM2_ESM.docx]

Meta-analysis of Epigenome Wide Association Studies of Major Depressive Disorder

Qingqin S Li^1,2^, Randall L Morrison^1,a^, Gustavo Turecki^3^, Wayne C Drevets^4^

^1^Neuroscience, Janssen Research & Development, LLC, Titusville, NJ, USA; ^2^JRD Data Science, Janssen Research & Development, LLC, Titusville, NJ, USA; ^3^Douglas Mental Health University Institute, McGill University, Montreal, Quebec, Canada; ^4^Neuroscience, Janssen Research & Development, LLC, La Jolla, CA, USA

^a^ Current affiliation: RLM Consulting LLC, 200 S Landmark Lane, Fort Washington, PA 19034

**Supplementary Figure S1**. QQ plots of EWAS in (A) cohort 1 (B) Cohort 2 (C) meta-analysis

**Supplementary Figure S2**. Manhattan plots (A) cohort 1, (B) cohort 2

**Supplementary Figure S3**. Association of cg01821149 annotated to *TNNT3* with MDD case control status in (A) cohort 1, (B) cohort 2.

**Supplementary Figure S4.** Correlation of effect size between cohort 2 and Generation Scotland penalized regression coefficient derived using wave 1 training data.

**Supplementary Figure S5**. DMR annotated to *IL17RA.*

**Supplementary Figure S1**. QQ plots of EWAS in (A) cohort 1 (B) Cohort 2 (C) meta-analysis

1. Cohort 1 (lambda = 1.048)


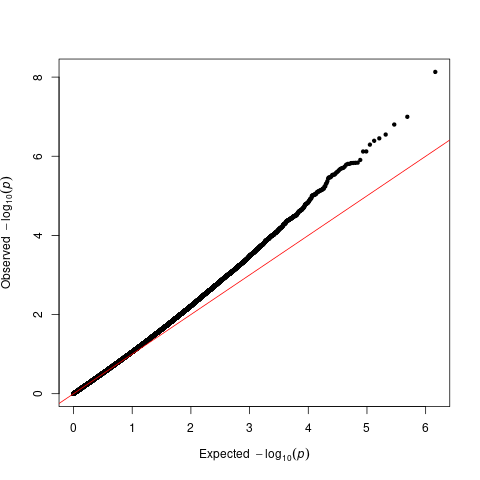


1. Cohort 2 (lambda = 1. 115)


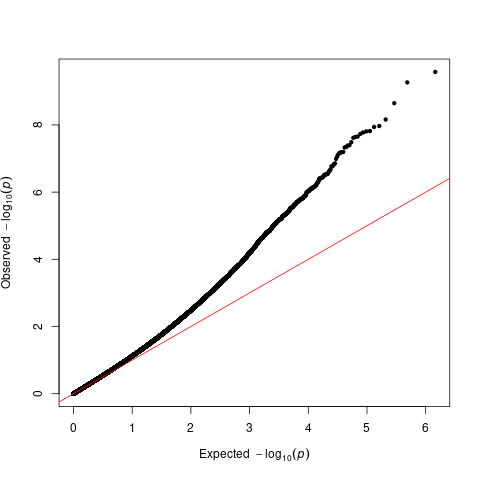


1. Meta-analysis (lambda = 0.812)


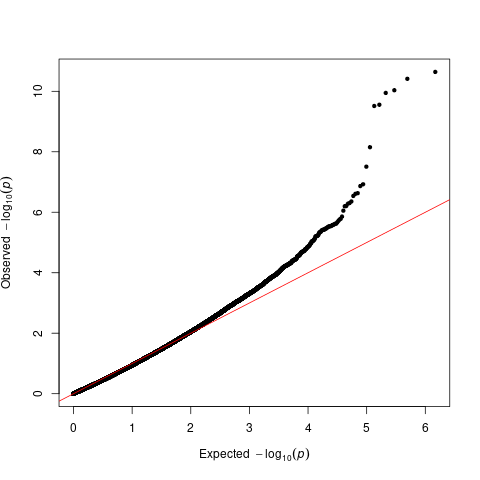


**Supplementary Figure S2**. Manhattan plots (A) cohort 1, (B) cohort 2

1. **Cohort 1**

1. **Cohort 2**

**Supplementary Figure S3**. Association of cg01821149 annotated to *TNNT3* with MDD case control status in (A) cohort 1, (B) cohort 2.

**Supplementary Figure S4.** Correlation of effect size between cohort 2 and Generation Scotland penalized regression coefficient derived using wave 1 training data [1].


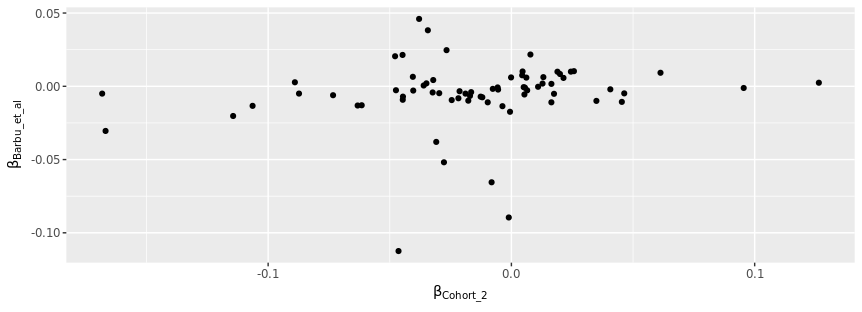


**Supplementary Figure S5**. DMR annotated to *IL17RA*.
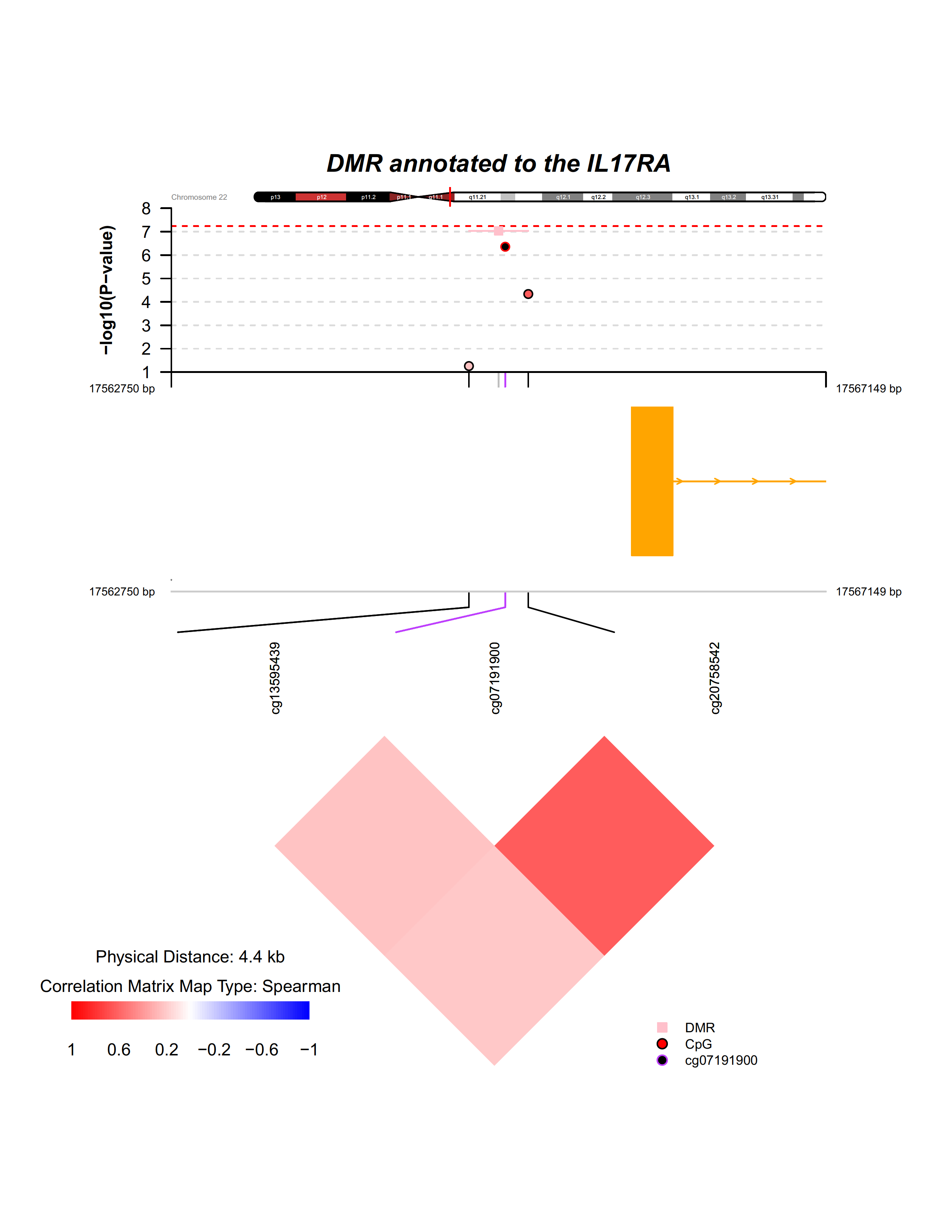


**Reference**

1. Barbu MC, Huider F, Campbell A, Amador C, Adams MJ, Lynall ME, Howard DM, Walker RM, Morris SW, Van Dongen J *et al*: **Methylome-wide association study of antidepressant use in Generation Scotland and the Netherlands Twin Register implicates the innate immune system**. *Molecular psychiatry* 2021.
